# Diagnostic performance of imaging modalities in chronic pancreatitis: a systematic review and meta-analysis

**DOI:** 10.1007/s00330-016-4720-9

**Published:** 2017-01-27

**Authors:** Y. Issa, M. A. Kempeneers, H. C. van Santvoort, T. L. Bollen, S. Bipat, M. A. Boermeester

**Affiliations:** 1 Department of Surgery, Academic Medical Centre, Meibergdreef 9, 1100DD Amsterdam, The Netherlands; 2 Department of Radiology, St Antonius Ziekenhuis, Koekoekslaan 1, 3430EM Nieuwegein, The Netherlands; 3 Department of Radiology, Academic Medical Centre, Meibergdreef 9, 1100DD Amsterdam, The Netherlands

**Keywords:** Chronic pancreatitis, Diagnostic imaging, Diagnostic accuracy, Meta-analysis

## Abstract

**Objectives:**

Obtain summary estimates of sensitivity and specificity for imaging modalities for chronic pancreatitis (CP) assessment.

**Methods:**

A systematic search was performed in Cochrane Library, MEDLINE, Embase and CINAHL databases for studies evaluating imaging modalities for the diagnosis of CP up to September 2016. A bivariate random-effects modeling was used to obtain summary estimates of sensitivity and specificity.

**Results:**

We included 43 studies evaluating 3460 patients. Sensitivity of endoscopic retrograde cholangiopancreatography (ERCP) (82%; 95%CI: 76%-87%) was significant higher than that of abdominal ultrasonography (US) (67%; 95%CI: 53%-78%; P=0.018). The sensitivity estimates of endoscopic ultrasonography (EUS), magnetic resonance imaging (MRI), and computed tomography (CT) were 81% (95%CI: 70%-89%), 78% (95%CI: 69%-85%), and 75% (95%CI: 66%-83%), respectively, and did not differ significantly from each other. Estimates of specificity were comparable for EUS (90%; 95%CI: 82%-95%), ERCP (94%; 95%CI: 87%-98%), CT (91%; 95% CI: 81%-96%), MRI (96%; 95%CI: 90%-98%), and US (98%; 95%CI: 89%-100%).

**Conclusions:**

EUS, ERCP, MRI and CT all have comparable high diagnostic accuracy in the initial diagnosis of CP. EUS and ERCP are outperformers and US has the lowest accuracy. The choice of imaging modality can therefore be made based on invasiveness, local availability, experience and costs.

***Key Points*:**

• *EUS, ERCP, MRI and CT have high diagnostic sensitivity for chronic pancreatitis*

• *Diagnostic specificity is comparable for all imaging modalities*

• *EUS and ERCP are outperformers and US has the lowest accuracy*

• *The choice of imaging can be made based on clinical considerations*

## Introduction

Chronic pancreatitis (CP) is a disabling inflammatory disease of the pancreas characterized by severe recurrent or continuous abdominal pain and considerable impact on the quality of life [1–4]. Patients with CP usually develop endocrine and exocrine insufficiency during the course of the disease as a result of the progressive loss of pancreatic parenchyma.

There is lack of international consensus regarding the initial diagnosis of CP, particularly at its early stages. The diagnosis is often made by a combination of clinical symptoms (e.g. abdominal pain, malabsorption, diabetes mellitus), pancreatic function tests (e.g. fecal elastase-1) and morphological abnormalities seen on imaging (e.g. calcifications, ductal lesions, pseudocysts) [5, 6]. Imaging plays a key role in the diagnosis and therapeutic management of patients with CP. The most frequently used imaging modalities for CP are endoscopic ultrasonography (EUS), endoscopic retrograde cholangiopancreatography (ERCP), magnetic resonance imaging (MRI), computed tomography (CT) and ultrasonography (US).

The aim of this meta-analysis was to determine the diagnostic accuracy of imaging modalities for the initial diagnostic assessment of CP.

## Methods

### Search

A search was performed in Cochrane Library, MEDLINE, EMBASE and CINAHL databases, without restrictions for publication date or language up to September 2016. The search included terms for chronic pancreatitis, EUS, ERCP, MR imaging, CT and US. For detailed search details, see Appendix Table 5.

### Selection of studies

All search hits were screened on title and abstract and eligible articles on full text by two reviewers independently (YI and MAK). Disagreements were solved through discussion with a third reviewer (MAB). Studies were eligible when EUS, ERCP, MR imaging, CT or US was evaluated in patients with suspected CP. Duplicates, reviews, letters, case reports and book chapters were excluded. The remaining studies were potentially eligible and their full text was retrieved. To identify additional relevant studies, the reference lists of the included studies were checked manually. Studies were included if they met the following criteria: (1) sufficient data was reported to construct 2 × 2 tables (true positive, false positive, true negative and false negative); (2) the imaging technique was compared with a reference standard (e.g. surgery, histology, follow-up). Exclusion criteria were: (1) evaluation of imaging techniques other than the aforementioned (e.g. PET-CT, EUS-FNA, EUS-elastography); (2) imaging techniques used for treatment of patients with CP (e.g. therapeutic ERCP, EUS-guided pseudocyst drainage); (3) in vitro studies; (4) studies that included less than five patients with CP; (5) studies where no separate analysis were done for patients with CP; and (6) full-text articles that were not available or retrievable.

### Data extraction and critical appraisal

Data was extracted systematically from the included studies by using a structured study record form. The following study design and patient characteristics were extracted: name of the first author, country of origin, year of publication, name of journal, study design, total number of patients included, number of included patients with CP, median or mean age, the proportion of male patients, and the patient inclusion criteria.

Data was extracted regarding the imaging characteristics: type of imaging modality, scoring criteria, technical features for each modality, and reported observer experience. Also data on the reference standard was extracted, such as clinical follow-up, surgery and histology.

The methodological quality of the included articles was assessed by the Quality Assessment of Diagnostic Accuracy Studies version 2 (QUADAS-2) tool [7]. The QUADAS-2 tool evaluates the risk of bias in four domains (patient selection, index test, reference standard, flow and timing) and the clinical applicability in the first three domains. Signaling questions were used to help assess the risk of bias and applicability. Possible answers were ‘yes’, ‘no’ or ‘unclear’ in which ‘yes’ indicates no risk of bias. In addition the GRADE scoring system for diagnostic tests was used, which assesses the quality of evidence for each imaging modality [8, 9]. Although the criteria are applicable to diagnostic test accuracy, the methods are less well established compared to interventional studies [10]. Two reviewers independently (YI and MAK) assessed the QUADAS-2 and the GRADE scoring system and all disagreements were resolved by reaching consensus.

### Data analysis

#### Overall diagnostic accuracy

For each included study we constructed a 2 × 2 contingency table for each imaging modality. If diagnostic accuracy was compared between different observers, mean values were calculated. Sensitivity and specificity estimates, the positive predictive value and negative predictive values, and the accuracy were calculated from the reconstructed contingency tables. We used the *I* ^2^ test with 95% confidence interval (95% CI) to quantify heterogeneity [11]. Mean logit sensitivity and specificity were acquired, and the anti-logit transformation was then obtained to calculate summary estimates of sensitivity and specificity with 95% CIs. Forest plots were made to visualize the sensitivity and specificity with the 95% CIs. Summary estimates of sensitivity and specificity, including 95% CI, were obtained by using a random-effects model [12]. In cases where a negative covariance between the logit sensitivity and logit specificity was obtained, summary receiver operating characteristic curve (sROC) were generated for each separate imaging modality. We used the *z* test to evaluate differences in sensitivity and specificity between the five imaging modalities. A *p* value of less than 0.05 indicated a statistically significant difference.

#### Heterogeneity exploration

The following factors were incorporated in the bivariate model and we evaluated the effect on the sensitivity and specificity, and cause of heterogeneity for all imaging modalities according to the QUADAS-2 tool: clear description of criteria for bias (low bias versus high bias or unclear) for (a) patient selection, (b) criteria for the index test used, (c) sufficient description and verification with the reference standard, and (d) the flow and timing.

#### Head to head comparison

A head to head comparison was performed in studies that compared the diagnostic accuracy of two or more imaging modalities. Heterogeneity was quantified by *I* ^2^ test, with 95% CI. The random-effects (*I* ^2^ > 25%) and fixed effects (*I* ^2^ ≤ 25%) models were used to obtain summary estimates of sensitivity and specificity, and compared with one another by a paired *z* test.

For data analysis, Review Manager (RevMan, version 5.3. Copenhagen: The Cochrane Collaboration, 2014) and SAS (version 9.3; SAS Institute, Cary, NC) were used. We adhered to the Preferred Reporting Items for Systematic Reviews and Meta-Analyses (PRISMA) guidelines [13].

## Results

### Study selection

**Fig. 1:**
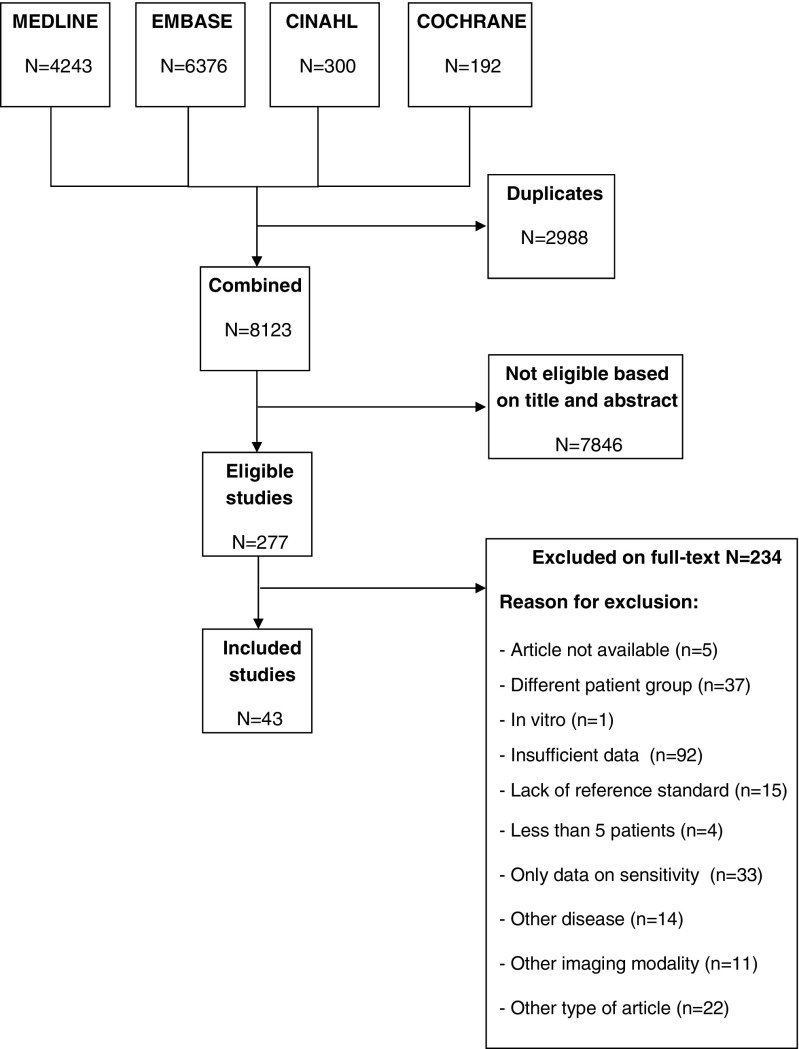
Flow chart

The initial search resulted in 11,111 hits, of which 2988 duplicates were removed, resulting in a total of 8123 titles and abstracts that were screened for eligibility. The full text of 277 articles was retrieved; 43 of these articles fulfilled the inclusion criteria. See Appendix Table 6 for the excluded articles. Figure 1 shows the flow chart of the search.

### Study and patient characteristics

Study characteristics, including the reference standard for the diagnosis of CP for each included study, are listed in Table 1. The 43 included studies were published between 1975 and 2016; 26 studies were prospective and 23 studies were published after the year 2000. A total of 3460 patients were evaluated, of which 1242 patients were diagnosed with CP [14–56]. The age of the patients ranged from 36 to 65 years, with a median of 50% male. Criteria for selection of patients were those with suspected pancreatic disease or patients with suspected CP. Patient characteristics are depicted in Table 2.

**Table 1:** Study characteristics of included studies

Study	Year	Country	P/R	OE	Modality	Reference standard for CP diagnosis
Adamek et al	2000	Germany	P	No	MRCP/ERCP	Histology (NA), FU (NA)
Albashir et al	2010	USA	R	Yes	EUS	Histology (all)
Alcaraz et al	2000	Spain	P	Yes	MRCP	Surgery (4), ERCP (70), PTC (7)
Balci et al	2006	USA and Germany	R	No	MRCP	ePFT (all)
Bolog et al	2004	Romania	R	No	MRCP	Surgery (NA), ERCP (NA), FU (NA)
Brand et al	2000	Germany	P	No	EUS	Histology (all)
Buscail et al	1995	France	P	No	US/CT/ERCP/EUS	Histology (7), morphological changes (i.e. calcifications) and exocrine insufficiency (42) + FU (all)
Catalano et al	1998	USA	P	No	EUS	ERCP + ePFT (all)
Chong et al	2007	USA	R	Yes	EUS	Surgery (all)
Conwell et al	2007	USA	R	Yes	EUS	ePFT (all)
Dramaix et al	1980	France	P	No	US/CT	Surgery (NA), ERCP (NA)
Fusari et al	2010	Italy	P	Yes	CT/MRCP	Biopsy (33), histology (7)
Gebel et al	1985	Germany	P	No	US/ERP	Obduction (NA), Surgery (NA), FU (NA)
Giovannini et al	1994	France	P	No	EUS	ERCP (all)
Glasbrenner et al	2000	Germany	P	Yes	EUS/ERCP	Surgery (all)
Gmelin et al	1981	Germany	P	No	US/CT/ERCP	Surgery (NA)+FU (NA)
Hellerhoff et al	2002	Germany	P	Yes	MRCP/sMRCP	ERCP (35), surgery (4), FU (56)
Imdahl et al	1999	Germany	P	Yes	CT	Histology (42), FU (6)
Kremer et al	1977	Germany	R	No	US	Clinical diagnosis (338), ERCP, surgery, ePFT, angiography (NA)
Lammer et al	1980	Germany	R	No	ERCP/CT	Surgery (31), angiography (16), clinical diagnosis (60)
Lawson et al	1978	USA	R	Yes	ERCP/US	Surgery (25), FU (50)
Lees et al	1979	UK	P	No	US	Surgery (36), ERCP (46)
Lin et al	1989	Taiwan	R	No	US/EUS	Histology (26), CT (4), surgery+ERCP (3)
Nattermann et al	1993	Germany	P	No	EUS	ERCP (94), FU (20)
Pamos et al	1998	Spain	P	Yes	MRCP	ERCP (all)
Parsi et al	2008	USA	R	Yes	ERCP	FU (all)
Pistolesi et al	1981	Italy	P	No	CT	Surgery (all)
Pungpapong et al	2007	USA	P	Yes	EUS	Clinical history, lab data, ERCP/CT/MRI and/or surgical pathology (all)
Pungpapong et al	2007	USA	P	Yes	MRCP/EUS	ERCP (48), surgery (9), FU (57)
Rudowicz-Pietruszewska et al	2002	Poland	P	No	MRCP	ERCP (all)
Sai et al	2008	Japan	P	Yes	sMRCP	ERCP (all)
Savarino et al	1980	Italy	R	No	CT	Surgery (NA), calcifications (NA), clinical and lab data (NA)
Scarabino et al	1989	Italy	R	No	ERCP, US, CT	Combination of CT, US and ERCP (all)
Schlaudraff et al	2008	USA and Germany	P	Yes	MRCP/sMRCP	Clinical history, laboratory, radiology (≥2 methods) (all)
Stevens et al	2009	USA	P	Yes	EUS	ePFT (all)
Sverko et al	2011	Croatia	R	No	MRCP	Histology (all)
Swobodnik et al	1983	Germany	P	No	US/CT/ERCP	FU (59), surgery (22)
Tox et al	2007	Germany	R	Yes	EUS	Surgery (79), FU (92)
Trikudanathan et al	2016	USA	R	YES	EUS	Histology (all)
Triller et al	1975	Switzerland	P	No	ERCP	Surgery (14), autopsy (1), FU (9)
Wiersema et al	1993	USA	P	No	EUS/ERCP	FU (51), ePFT (16)
Zhang et al	2003	USA	R	No	MRCP	US (12), CT (11), ERCP (6)
Zuccaro et al	2009	USA	R	No	MRCP/sMRCP	ePFT (all)

*P* prospective, *R* retrospective, *OE* observer experience reported, *PTC* percutaneous transhepatic cholangiogram, *ePFT* endoscopic pancreatic function test, *FU* follow-up, *NA* not available

**Table 2:** Patient characteristics of included studies

Study	Nr pts	Age	Male (%)	Nr pts CP	Patient selection
Adamek et al	124	55	61%	57	Suspected pancreatic mass (clinical presentation, lab, US)
Albashir et al	23	43*	57%	19	Suspected chronic pancreatitis (clinical presentation)
Alcaraz et al	81	65**	31%	8	Suspected pancreatobiliary disease (clinical presentation, US)
Balci et al	30	48*	17%	11	Suspected early CP (clinical presentation)
Bolog et al	103	57*	43%	15	Suspected pancreatobiliary disease (US/CT or clinical presentation)
Brand et al	115	61*	59%	24	Suspected focal pancreatic lesion (US/CT/ERCP or lab/tumour markers)
Buscail et al	62	50*	79%	44	Suspected chronic pancreatitis (clinical presentation, lab, imaging)
Catalano et al	80	51*	40%	38	Non-alcoholic recurrent acute pancreatitis (3–11 episodes)
Chong et al	71	45*	46%	64	Suspected chronic pancreatitis (clinical presentation)
Conwell et al	56	44*	45%	38	Suspected chronic pancreatitis (clinical presentation)
Dramaix et al	50	52*	66%	18	Suspected pancreatic disease (clinical presentation)
Fusari et al	40	62*	55%	8	Suspected pancreatic mass (clinical presentation and US)
Gebel et al	US: 56, ERP: 45	NA	NA	US: 22, ERP: 16	Suspected pancreatic disease (clinical presentation)
Giovannini et al	26	NA	NA	17	Suspected pancreatobiliary disease (clinical presentation, imaging/lab)
Glasbrenner et al	85	NA	NA	41	Suspected pancreatic mass (clinical presentation, US/CT)
Gmelin et al	41	54*	68%	19	Suspected pancreatic disease (clinical presentation)
Hellerhoff et al	95	NA	NA	26	Suspected pancreatic disease (clinical presentation)
Imdahl et al	48	58*	60%	12	Suspected pancreatic disease (clinical presentation)
Kremer et al	446	NA	NA	61	Suspected pancreatic disease (clinical presentation)
Lammer et al	107	NA	NA	39	Suspected pancreatic disease (clinical presentation)
Lawson et al	75	NA	NA	26	Suspected pancreatic disease (clinical presentation)
Lees et al	98	NA	NA	20	Suspected pancreatic disease (clinical presentation)
Lin et al	33	47*	58%	7	Suspected pancreatic disease (clinical presentation)
Nattermann et al	114	53*	67%	51	Suspected pancreatic disease (clinical presentation)
Pamos et al	41	64*	59%	5	Suspected pancreatobiliary disease (clinical presentation)
Parsi et al	35	46**	46%	24	Suspected chronic pancreatitis (clinical presentation)
Pistolesi et al	100	NA	NA	31	Suspected pancreatic disease (clinical presentation)
Pungpapong et al	79	50**	35%	38	Suspected chronic pancreatitis (clinical presentation)
Pungpapong et al	99	55**	41%	40	Suspected chronic pancreatitis (clinical presentation)
Rudowicz-Pietruszewska et al	88	52*	64%	9	Suspected pancreatobiliary disease (clinical presentation, lab, US/CT)
Sai et al	28	36*	NA	16	Mild chronic pancreatitis (ERCP)
Savarino et al	108	47**	67%	59	Suspected pancreatic disease (clinical presentation)
Scarabino et al	63	44**	63%	12	Suspected of biliopancreatic disease (clinical presentation)
Schlaudraff et al	62	NA	NA	9	Suspected chronic pancreatitis (clinical presentation)
Stevens et al	100	NA	38%	41	Suspected chronic pancreatitis (clinical presentation)
Sverko et al	29	44**	52%	14	Suspected pancreatic disease (clinical presentation)
Swobodnik et al	81	49*	52%	27	Suspected pancreatic disease (clinical presentation)
Tox et al	171	61*	NA	65	Suspected pancreatic disease (clinical presentation)
Trikudanathan et al	68	39*	18%	56	Total pancreatectomy for non-calcific chronic pancreatitis
Triller et al	24	52*	83%	11	Suspected pancreatobiliary disease (clinical presentation)
Wiersema et al	67	45*	20%	30	Suspected pancreatobiliary disease (clinical presentation)
Zhang et al	44	50*	30%	24	Suspected early or mild chronic pancreatitis (clinical presentation, US/CT/ERCP)
Zuccaro et al	69	43*	35%	28	Suspected chronic pancreatitis (clinical presentation)

*NA* not available

*Mean

**Median

**Fig. 2:**
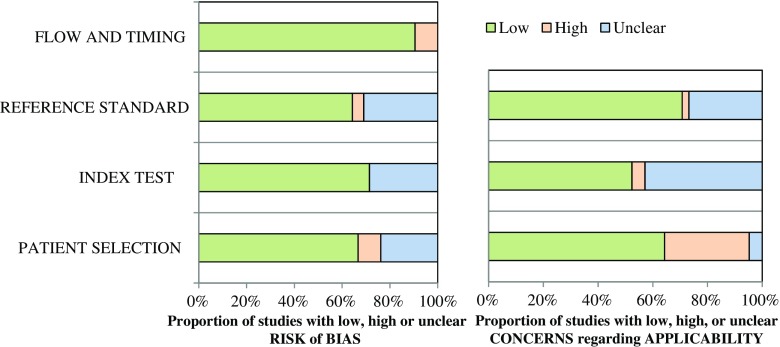
Summary of study quality (QUADAS-2)

The risk of bias, assessed by QUADAS-2, was low in 28% of the studies and high in 19% of the studies. The concerns about applicability were low in 30% of the studies and high in 40% of the studies. The QUADAS-2 characteristics for each domain are depicted in Fig. 2 and outlined for each study in Appendix Table 7. The quality of evidence for all five imaging modalities according to the GRADE scoring system was very low. The GRADE scores for each imaging modality and characteristics for each study are outlined in Appendix Tables 8 and 9.

EUS was the most frequently evaluated imaging modality; 16 studies including 1249 patients [15, 19–23, 27, 28, 36, 37, 41, 42, 48, 51, 53, 56]. ERCP was studied in 11 studies including 742 patients [14, 20, 26, 28, 29, 33, 34, 39, 46, 50, 52]; MRCP, including secretin-enhanced MRCP, was evaluated in 14 studies including 933 patients [14, 16–18, 25, 30, 38, 42–44, 47, 49, 54, 55]; CT in 10 studies including 700 patients [20, 24, 25, 29, 31, 33, 40, 45, 46, 50] and abdominal US in 10 studies which included 1005 patients [20, 24, 26, 29, 32, 34–36, 46, 50]. The imaging characteristics for each study and modality in an individual study are listed in Appendix Table 11. Three of the 43 articles reported about complications of the imaging modality used; these were complications related to ERCP (being post-ERCP pancreatitis) with a mean complication rate of 4% [14, 20, 28].

### Overall diagnostic accuracy

**Fig. 3:**
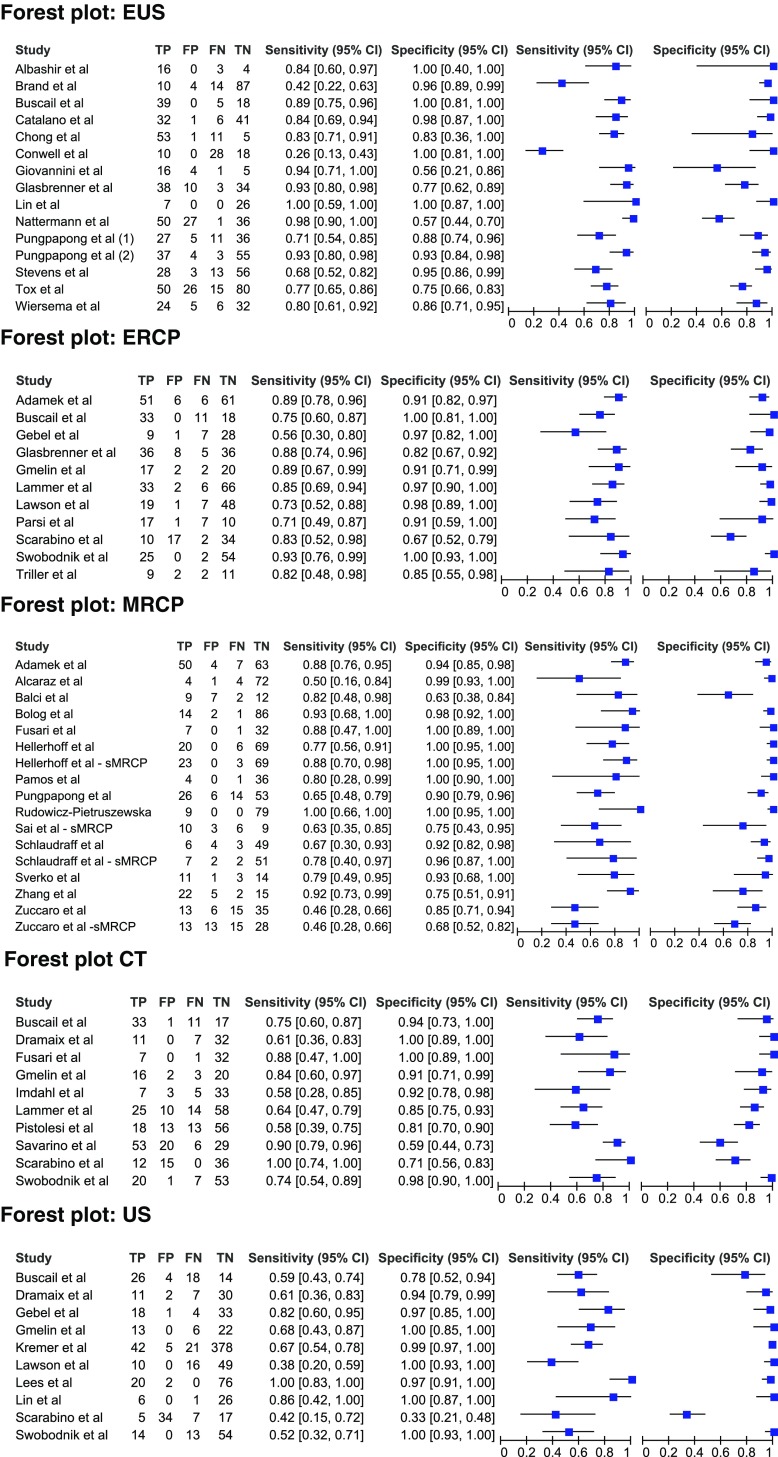
Forest plot for sensitivity and specificity

**Fig. 4:**
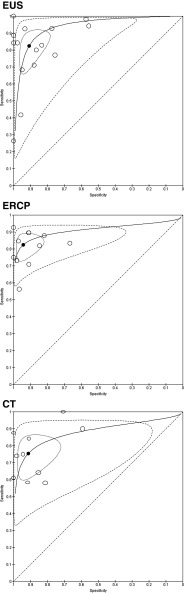
Receiver operator curves (ROC)

Analyses for summary estimates of sensitivity and specificity were done for EUS, ERCP, MRI, CT and US (Table 3). Figures 3 and 4 show sensitivity and specificity of individual studies in forest plots and in receiver operator curves (ROC), respectively. A negative covariance between the logit sensitivity and logit specificity was not obtained; therefore, no sROC for MRI and US could be drawn. The summary estimate of sensitivity for EUS, ERCP, MRCP, CT and US was 81%, 82%, 78%, 75% and 67%, respectively. The summary estimate of specificity for EUS, ERCP, MRCP, CT and US was 90%, 94%, 96%, 91% and 98%, respectively. Sensitivity of ERCP was significant higher than sensitivity of US (*p* = 0.018). Other pairwise comparisons of sensitivity between imaging modalities revealed no significant difference. Specificity did not differ significantly among all modalities (Table 3). Sensitivity and specificity values for each study are listed in Appendix Table 10.

**Table 3:** Estimated overall sensitivity, specificity and heterogeneity according to imaging modality

Modality	*N* studies	*N* patients	Sensitivity (95% CI)	Specificity (95% CI)	Heterogeneity (*I* ^2^)
EUS	16	1249	81% (70–89%)	90% (82–95%)	82%/73%
ERCP	11	742	82% (76–87%)	94% (87–98%)	39%/67%
MRCP	14	933	78% (69–85%)	96% (90–98%)	59%/65%
CT	10	700	75% (66–83%)	91% (81–96%)	50%/71%
US	10	1005	67% (53–78%)	98% (89–100%)	40%/93%

Random effects model

### Heterogeneity exploration

The bivariate model for heterogeneity exploration showed that the factor ‘flow and timing’ was significantly associated with a higher sensitivity of US (*p* = 0.01). ‘Description and verification with the reference standard’ was significantly associated with a higher specificity for MRCP (*p* = 0.0002).

### Head to head comparison

Six head to head comparisons were performed (Table 4). The specificity of ERCP and EUS, and the sensitivity of ERCP, EUS and CT in the summary estimates of the head to head studies were significantly higher as compared with US.

**Table 4:** Head to head comparison

Comparison	*N* studies	*N* patients	Modality	Sensitivity (95% CI)	Specificity (95% CI)
US vs ERCP^a^	6	423	US	57% (49–65%)	94% (74–99%)
			ERCP	78% (71–85%)	98% (89–100%)
US vs CT^b^	5	297	US	58% (49–66%)	77% (71–83%)
			CT	77% (68–83%)	82% (74–88%)
CT vs ERCP^b^	5	354	CT	75% (67–82%)	86% (81–90%)
			ERCP	84% (77–89%)	90% (85–93%)
EUS vs ERCP^b^	3	214	EUS	88% (80–93%)	85% (76–91%)
			ERCP	86% (78–91%)	92% (85–96%)
MRCP vs sMRCP^b^	3	226	MRCP	62% (49–73%)	94% (89–97%)
			sMRCP	68% (56–79%)	91% (85–94%)
EUS vs US^b^	2	95	EUS	90% (82–98%)	100%
			US	63% (49–76%)	91% (82–99%)

Sensitivity: US vs ERCP (*p* < 0.001), US vs CT (*p* = 0.002), EUS vs US (*p* = 0.001)

Specificity: US vs ERCP (*p* = 0.003), EUS vs US (*p* = 0.04)

^a^Random effects model

^b^Fixed effects model

The head to head comparison of US versus ERCP comparison yields a sensitivity of 57% (49–65%) versus 78% (71–85%) (*p* < 0.001); and a specificity of 94% (74–99%) versus 98% (89–100%) (*p* = 0.003), respectively [20, 26, 29, 34, 46, 50]. The comparison between US and CT yields a sensitivity of 58% (49–66%) and 77% (68–83%) (*p* = 0.002), respectively [20, 24, 29, 46, 50]. And finally, the comparison of EUS versus US comparison yields a sensitivity of 90% (82–98%) versus 63% (49–76%) (*p* = 0.001); and a specificity of 100% versus 91% (82–99%) (*p* = 0.04), respectively [20, 36]. There were no significant differences in the sensitivity and specificity estimates between ERCP and EUS [20, 28, 53], MRCP and sMRCP [30, 47, 55] or ERCP and CT [20, 29, 33, 46, 50]. The heterogeneity (*I* ^2^) between US and ERCP (>25%) was higher (>25%) than in the other comparisons (*I* ^2^ ≤ 25%).

## Discussion

EUS, ERCP, MRI and CT all have comparable high diagnostic accuracy in the initial diagnosis of chronic pancreatitis. EUS and ERCP are outperformers and US has the lowest accuracy. The choice of imaging modality can therefore be made on the basis of invasiveness, local availability, experience and costs.

Several recent guidelines [57–59] advocate the use of EUS, MRCP or CT for the diagnosis of CP, although summary estimates of their accuracy, thus far, were lacking. There is one guideline from Germany on CP that has reported sensitivity and specificity regarding EUS, ERCP, MRCP and US, although not for CT [60]. In this guideline 14 studies were selected, reporting ranges rather than pooling the data on sensitivity and specificity estimates. This method resulted in results slightly different from those in the present meta-analyses. For example the guideline reports a sensitivity of 70–80% for ERCP and 88% for MRI versus summary estimates of 82% and 78%, respectively, in the present meta-analyses. The European Society of Radiology (ESR) is developing the ESR iGuide, a clinical decision support system for European imaging referral guidelines, covering various clinical scenarios, indications and recommendations (www.esriguide.org) [61–63]. The results from the present systematic review may be useful to incorporate in that system.

We excluded three studies where sensitivity and specificity data were provided, but it was not possible to extract sufficient data to produce 2 × 2 tables and calculate the diagnostic accuracy values, because only the sensitivity and specificity estimates were given [64–66]. In the study by Wang et al., estimates of sensitivity and specificity for EUS, ERCP and US were in line with the present results; the sensitivity of MR imaging and CT, however, were much lower (66% and 61%) [66]. The studies by Clave et al. and Orti et al. showed a lower sensitivity of ERCP (62% and 70%, respectively) compared to present results (82%) [64, 65].

The risk of missing important studies was minimized by performing a search in four major databases by two reviewers independently, without setting any restrictions for language and publication date. However, this systematic review has some limitations. The heterogeneity of the pooled studies was moderate to high in all analyses (between 39% and 93%). However, in the head to head comparison analyses, the heterogeneity was low in most comparisons (<25%). Furthermore, the heterogeneity of the reference standards used in the studies could have influenced individual study results. Surgery, histology and long-term follow-up of patients are reliable methods. Some reference standards, such as the use of endoscopic pancreatic function test (ePFT) for establishing the diagnosis of CP, could have resulted in under- or overestimation of the sensitivity and specificity. In addition, the diagnosis of CP and the criteria used are different in different stages of the disease (e.g. absence of calcifications in the early phase of the disease). Another limitation was that our analyses included imaging studies and imaging protocols performed over the last 40 years in different centres with inherent variations in techniques and equipment. Especially in the last decade the quality of some imaging modalities (e.g. MRCP and CT) has improved considerably. Also there were concerns about the quality of the available evidence, as assessed by QUADAS-2 and the GRADE scoring system.

The highest scores for accuracy in the diagnosis of CP were found for EUS and ERCP, but these are invasive techniques. ERCP has a relatively high risk of complications, such as post-ERCP pancreatitis (1.6–15.7%, mean complication rate of 4%) and is nowadays only used for therapeutic purposes (e.g. stenting of pancreatic duct) [67–69]. To date, diagnostic ERCP is largely replaced by EUS and the cross-sectional imaging modalities CT and MRCP.

It has been suggested that CT is better in detecting parenchymal calcifications and intraductal calcifications compared to MRCP [70–73]. On the other hand, MRCP is more often able to detect significant abnormalities of the pancreatic duct (e.g. PD dilatation and strictures) and slight changes of the pancreatic parenchyma and side branches, which can be attributed to early signs CP (i.e. atrophy, side branch ectasia) compared to CT [74]. Early diagnosis can also lead to a timely start of treatment, which has been associated with improved long-term outcome [75]. Nevertheless, for very early CP this association needs to be established in further research, such as the ESCAPE trial, evaluating the effect of early intervention in patients with CP [76]. As diagnostic sensitivity of CT and MRCP is not significantly lower than that of ERCP and EUS, and specificity is comparable, non-invasive modalities except for US are a likely first choice in patients with suspected pancreatic disease including chronic pancreatitis.

## APPENDIX

Table 5

**Table 5:** Search terms

	MeSH terms	All Fields
Chronic pancreatitis	Pancreatitis, chronic [MeSH]	Chronic pancreatitis [All Fields]
AND		
EUS	Endosonography [MeSH]	EUS [All Fields]
OR		
ERCP	Cholangiopancreatography, Endoscopic Retrograde [MeSH]	Endoscopic Retrograde Cholangiopancreatograp* [All Fields] OR ERCP [All Fields]
OR		
MRCP	Magnetic Resonance Imaging [MeSH] OR Cholangiopancreatography, Magnetic Resonance [MeSH]	Magnetic resonance imaging [All Fields] OR MRI [All Fields] OR MRCP [All Fields] OR Magnetic Resonance Cholangio* [All Fields]
OR		
sMRCP		Magnetic Resonance Imaging [All Fields] AND secretin [All Fields] OR sMRI [All Fields]
OR		
CT	Tomography, X-Ray Computed [MeSH]	(Tomography [All Fields] AND x-ray [All Fields] AND computed [All Fields]) OR Computed Tomography [All Fields]) OR CT scan* [All Fields]
OR		
US	Ultrasonography [MeSH]	Ultrasonogra* [All Fields] OR ultrasound [All Fields]

*MeSH* Medical Subject Headings

Table 6

**Table 6:** Excluded articles based on full text

Author	Year	Journal	Reason for exclusion
Borsukov et al	2001	Ross Gastroenterol Zh	Article not available
Diad'kin et al	2013	Vestnik rentgenologii i radiologii	Article not available
Dotsenko et al	1985	Vrach Delo	Article not available
Rosch et al	1989	Z Gastroenterologie	Article not available
Suzdalev et al	1992	Likars'ka sprava	Article not available
Agarwal et al	2008	GIE	Exclusive patient group
Brailski et al	1989	Vutr Boles	Exclusive patient group
Brailski et al	1984	Vutr Boles	Exclusive patient group
Brimiene et al	2011	Medicina	Exclusive patient group
Carlucci et al	1989	HPB Surgery	Exclusive patient group
Chowdhury et al	2005	Pancreas	Exclusive patient group
Cotton et al	1980	Radiology	Exclusive patient group
DelMaschio et al	1991	Radiology	Exclusive patient group
Erturk et al	2006	Am J Gastroenterol	Exclusive patient group
Frick et al	1982	Gastrointest Rad	Exclusive patient group
Gheonea et al	2013	BMC Gastroenterology	Exclusive patient group
Goodale et al	1981	Ann Surg	Exclusive patient group
Hanninen et al	2002	Radiology	Exclusive patient group
Hatano et al	1998	Nippon rinsho J	Exclusive patient group
Hocke et al	2008	Dtsch Med Wochenschr	Exclusive patient group
Hocke et al	2006	WJG	Exclusive patient group
Hocke et al	2012	Z Gastroenterologie	Exclusive patient group
Huang et al	2011	J Dig dis	Exclusive patient group
Imbriaco et al	2006	Radiol Med	Exclusive patient group
Kawai et al	2012	Eur J Rad	Exclusive patient group
Kim et al	2007	J MRI	Exclusive patient group
Kursawa et al	1991	Radiol Diagn	Exclusive patient group
Lu et al	2013	Acad J Sec Mil Med University	Exclusive patient group
Lutz et al	1975	Klin Wschr	Exclusive patient group
Morris-Stiff et al	2009	J Pancreas	Exclusive patient group
Papp et al	1978	Wiener klin Wchnschrft	Exclusive patient group
Pomerri et al	1991	Radiologia Med	Exclusive patient group
Rosch et al	2000	Am J Gastroenterol	Exclusive patient group
Sandrasegaran et al	2013	AJR	Exclusive patient group
Sendler et al	2000	World J Surg	Exclusive patient group
Sugumar et al	2011	Gut	Exclusive patient group
Testoni et al	1981	Acta Endoscopica	Exclusive patient group
Tiushin et al	2003	Voprosy onkologii	Exclusive patient group
Varadarajulu et al	2007	GIE	Exclusive patient group
Viceconte et al	1980	Ann ital chir	Exclusive patient group
Yamada et al	2010	Abdom Imaging	Exclusive patient group
Zhu et al	2013	PLOS one	Exclusive patient group
Bhutani et al	2009	Pancreas	In vitro
Akisik et al	2013	AJR	No diagnostic values for CP
Alempijević et al	2005	Vojnosanit Pregl	No diagnostic values for CP
Alpern et al	1985	Radiology	No diagnostic values for CP
Ardelean et al	2014	Med Ultrason	No diagnostic values for CP
Ardengh et al	2011	GIE	No diagnostic values for CP
Ascunce et al	2010	Surg End	No diagnostic values for CP
Baert et al	1977	Radiologe	No diagnostic values for CP
Balci et al	2010	J MRI	No diagnostic values for CP
Beliao et al	2012	Eur J Rad	No diagnostic values for CP
Bender et al	1999	Invest Rad	No diagnostic values for CP
Bhatt et al	2005	Indian J Rad Imag Ass	No diagnostic values for CP
Bonanno et al	1994	Giorn Ital End Dig	No diagnostic values for CP
Bruhlmann et al	1976	RoFo	No diagnostic values for CP
Caletti et al	1982	British j Surgery	No diagnostic values for CP
Cao	1989	Zhonghua yi xue za zhi	No diagnostic values for CP
Cappeliez et al	2000	Radiology	No diagnostic values for CP
Chang et al	2010	GIE	No diagnostic values for CP
Cohen et al	2014	Dig Dis Sci	No diagnostic values for CP
Concia et al	2014	Invest Rad	No diagnostic values for CP
Dale et al	1979	Electromedica	No diagnostic values for CP
Das et al	2008	GIE	No diagnostic values for CP
Delbeke et al	1999	J Nucl Med	No diagnostic values for CP
Dite et al	1982	Vnitrni Lekarstvi	No diagnostic values for CP
Dronamraju et al	2016	Ann Gastroenterol	No diagnostic values for CP
D’Souza et al	2015	Dig Dis Sci	No diagnostic values for CP
Eitner et al	1979	Dtsch Zeitschr Verdauungs- und Stoffwechselkrankheiten	No diagnostic values for CP
Eloubeidi et al	2013	Pancreas	No diagnostic values for CP
Ergul et al	2014	Rev Esp Med Nucl Im Mol	No diagnostic values for CP
Ferrucci et al	1979	Radiology	No diagnostic values for CP
Foley et al	1980	Gastrointest Rad	No diagnostic values for CP
Fontana et al	1976	Gut	No diagnostic values for CP
Foster et al	1984	BMJ	No diagnostic values for CP
Gardner et al	2014	Pancreas	No diagnostic values for CP
Gincul et al	2014	Endoscopy	No diagnostic values for CP
Gowland et al	1981	Lancet	No diagnostic values for CP
Grant et al	1981	J Am Osteopathic Ass	No diagnostic values for CP
Harada et al	1977	Gastroenterologica Jap	No diagnostic values for CP
He et al	2014	Pancreas	No diagnostic values for CP
Hoki et al	2009	J Gastroenterol	No diagnostic values for CP
Hollerbach et al	1994	Med Klinik	No diagnostic values for CP
Horii et al	1982	Jap J Gastroenterol	No diagnostic values for CP
Johnson et al	1999	Radiology	No diagnostic values for CP
Jones et al	1988	Clin Radiol	No diagnostic values for CP
Kamisawa et al	2007	J Gastroenterol	No diagnostic values for CP
Kersting et al	2009	Gastroenterology	No diagnostic values for CP
Kitano et al	2004	Gut	No diagnostic values for CP
Laghi et al	1998	Chirurgia	No diagnostic values for CP
Leblanc et al	2014	Pancreas	No diagnostic values for CP
Leblanc et al	2014	Pancreas	No diagnostic values for CP
Li et al	2001	Zhongguo yi xue ke xue	No diagnostic values for CP
Loginov et al	1976	Sovetskaya Meditsina	No diagnostic values for CP
Lopez et al	2002	Radiology	No diagnostic values for CP
Manfredi	2000	Radiology	No diagnostic values for CP
Modder et al	1979	RoFo	No diagnostic values for CP
Montori et al	1979	Min Diet Gastroent	No diagnostic values for CP
Napoleon et al	2010	Endoscopy	No diagnostic values for CP
Novis et al	1976	S Afr Med J	No diagnostic values for CP
Ohtsubo et al	2008	Gastroenterolog Endoscopy	No diagnostic values for CP
Orlikov et al	2007	Ter Arkh	No diagnostic values for CP
Park et al	2008	The Korean J Gastroenter	No diagnostic values for CP
Petersein et al	2002	RoFo	No diagnostic values for CP
Pezzelli et al	2013	Pancreas	No diagnostic values for CP
Pomerri et al	1987	Radiologia Med	No diagnostic values for CP
Rickes et al	2002	Scand J Gastroenterol	No diagnostic values for CP
Rosenberger et al	1979	MMW	No diagnostic values for CP
Russell et al	1978	Gut	No diagnostic values for CP
Sahai et al	1998	GIE	No diagnostic values for CP
Sainani et al	2009	AJG	No diagnostic values for CP
Sica et al	2002	J MRI	No diagnostic values for CP
Sica et al	1999	Radiology	No diagnostic values for CP
Songur et al	2000	Digest Endoscopy	No diagnostic values for CP
Stevens et al	2010	WJG	No diagnostic values for CP
Struve et al	1982	Diagnostik & Intensivtherapie	No diagnostic values for CP
Sun et al	2010	Acad J Sec Mil Med University	No diagnostic values for CP
Tamura et al	2006	Radiology	No diagnostic values for CP
Tellez-Avila et al	2014	WJG	No diagnostic values for CP
Tirkes et al	2016	J MRI	No diagnostic values for CP
Trikudanathan et al	2015	Am J Gastroenterol	No diagnostic values for CP
Tripathi et al	2002	Indian J Gastroenterol	No diagnostic values for CP
Tympner et al	1979	Leber Magen Darm	No diagnostic values for CP
Tympner et al	1977	Verhand Dtschen Gesellschaft fur Innere Medizin	No diagnostic values for CP
Uskudar et al	2009	Pancreas	No diagnostic values for CP
Valentini et al	1981	Endoscopy	No diagnostic values for CP
Varghese et al	2002	Clin Radiol	No diagnostic values for CP
Wang et al	2013	WJG	No diagnostic values for CP
Wierzbicka-Paczos et al	1998	Gastroenterologia Polska	No diagnostic values for CP
Wierzbicka-Paczos et al	1999	Polski Merk Lek	No diagnostic values for CP
Will et al	2010	Ultraschall Med	No diagnostic values for CP
Zaheer et al	2014	Eur J Rad	No diagnostic values for CP
Bian et al	2014	Chin J Radiol	No reference standard
Braganza et al	1978	Clin Radiol	No reference standard
Gillams et al	2007	Eur J Rad	No reference standard
Helmberger et al	2000	RoFo	No reference standard
Hernandez Garces et al	2004	J Pancreas	No reference standard
Ho et al	2006	Clin Gastroenterol Hep	No reference standard
Kalmar et al	1984	Southern Medical J	No reference standard
Kalmin et al	2011	Can J Gastroenterol	No reference standard
Kaufman et al	1989	GIE	No reference standard
Kumon et al	2012	GIE	No reference standard
Manfredi et al	1998	La Rad Medica	No reference standard
Novotny et al	2000	Bratisl Lek Listy	No reference standard
Ponette et al	1976	Acta Gastro-Enterol Belgica	No reference standard
Sanyal et al	2012	AJR	No reference standard
Yoshimoto et al	1980	Jap J Gastroenterol	No reference standard
Grossjohann et al	2010	Scand J Gastroenterol	Not enough patients
Sood et al	1992	Indian J Gastroenterol	Not enough patients
Zhi et al	2002	Chin J Digestive Dis	Not enough patients
Zhong et al	2003	WJG	Not enough patients
Ainsworth et al	2003	Endoscopy	Only sensitivity reported
Bastid et al	1995	J d'Echographie et de Med par Ultrasons	Only sensitivity reported
Campisi et al	2009	Clin Radiol	Only sensitivity reported
Dancygier et al	1986	Scand J Gastroenterol	Only sensitivity reported
Giday et al	2011	J Gastr Hep	Only sensitivity reported
Guarita et al	1982	AMB	Only sensitivity reported
Guo et al	2003	Chin J Digestive Dis	Only sensitivity reported
Kahl et al	2002	GIE	Only sensitivity reported
Kim et al	2001	AJR	Only sensitivity reported
Kolmannskog et al	1981	Acta Radiologica	Only sensitivity reported
Lackner et al	1980	RoFo	Only sensitivity reported
Lawson	1978	Radiology	Only sensitivity reported
Manfredi	2002	Radiology	Only sensitivity reported
Mao et al	2011	WCJD	Only sensitivity reported
Nakashio	1992	Acta medica	Only sensitivity reported
Noguchi et al	1985	Gastroenterolog Endoscopy	Only sensitivity reported
Propp	2011	Vestnik khirurgii imeni	Only sensitivity reported
Rossi et al	1996	Giorn Ital End Dig	Only sensitivity reported
Sahel et al	1976	Acta Endoscopica	Only sensitivity reported
Seicean et al	2010	Ultraschall Med	Only sensitivity reported
Sildiroglu	1985	Rontgenpraxis	Only sensitivity reported
Singh et al	1993	Indian J Rad Imag	Only sensitivity reported
Sivak et al	1986	Scand J Gastroenterol	Only sensitivity reported
Stabile Ianora et al	2013	Recenti Prog Med	Only sensitivity reported
Stevens et al	2008	Dig Dis Sci	Only sensitivity reported
Stevens et al	2010	Dig Dis Sci	Only sensitivity reported
Triller et al	1983	Computertomographie	Only sensitivity reported
Uchida et al	1997	Jap J Clin Radiology	Only sensitivity reported
Vitale et al	2009	The Am Surgeon	Only sensitivity reported
Wang et al	2009	J Gastr Hep	Only sensitivity reported
Wu et al	2006	World Chin J Dig	Only sensitivity reported
Yanling et al	2001	Chinese J Gastroenterol	Only sensitivity reported
Zhou et al	1993	Zhonghua nei ke za zhi	Only sensitivity reported
Aithal et al	2002	GIE	Other disease
Doust et al	1976	Radiology	Other disease
Engjom et al	2015	Scan J Gastroenterol	Other disease
Huang et al	2009	Acad J Sec Mil Med University	Other disease
Kushnir et al	2011	GIE	Other disease
Lai et al	2004	Endoscopy	Other disease
Leblanc et al	2014	Pancreas	Other disease
Matos et al	2001	GIE	Other disease
Mosler et al	2012	Dig Dis Sci	Other disease
Novis et al	2010	Rev Colegio Brasileiro Cirurg	Other disease
Rana et al	2012	J Gastr Hep	Other disease
Ranney et al	2012	GIE	Other disease
Sainani et al	2015	Pancreas	Other disease
Soto et al	2005	Radiology	Other disease
Akisik et al	2009	Radiology	Other imaging modality
Cherian et al	2010	HPB Surgery	Other imaging modality
Glaser et al	1994	Int J Pancreatology	Other imaging modality
Glaser et al	1989	Scand J Gastroenterol	Other imaging modality
Glaser et al	1985	Ultraschall Med	Other imaging modality
Hocke et al	2007	Pancreas	Other imaging modality
Kumon et al	2010	GIE	Other imaging modality
Saftoiu et al	2008	GIE	Other imaging modality
Sreenarasimhaiah	2008	J Clin Gastroenterol	Other imaging modality
Tummula et al	2013	Clin Transl Gastroenterol	Other imaging modality
Uehara et al	2011	J Gastr Hep	Other imaging modality
Abdalla et al	2012	Gastroenterolgy	Other type of article
Arsac et al	1981	Med Chirurgie Digest	Other type of article
Ashida et al	2011	J Gastr Hep	Other type of article
Chvatalova et al	2012	Pancreatology	Other type of article
Czako et al	2007	J Gastroenterol	Other type of article
Gupta et al	2013	JIMSA	Other type of article
Heverhagen et al	2007	RoFo	Other type of article
Kasugai et al	1982	Stomach and intestine	Other type of article
Kent et al	2008	Pancreas	Other type of article
Markwardt et al	1980	Radiologia Diagn	Other type of article
Munoz et al	2010	Rev Med de Chile	Other type of article
Musunuri et al	2015	Ind J Gastroenterol	Other type of article
Quinn et al	2012	Gut	Other type of article
Romagnuolo et al	2012	GIE	Other type of article
Sherman et al	2012	GIE	Other type of article
Shibukawa et al	2015	Dig Endos	Other type of article
Stevens et al	2008	Pancreas	Other type of article
Takahashi et al	2014	AJR	Other type of article
Trus et al	1998	Probl Gen Surg	Other type of article
Vadrot et al	1981	Med Chirurgie Digest	Other type of article
Zaruba et al	2012	Pancreatology	Other type of article
Zhang et al	2011	J Gastr Hep	Other type of article

Table 7

**Table 7:** QUADAS-2 characteristics for each study

Study	Bias				Applicability		
	Patient selection	Index test	Reference standard	Flow and timing	Patient selection	Index test	Reference standard
Adamek et al	Low	Low	Low	Low	Unclear	Unclear	Low
Albashir et al	Low	Low	Low	Low	Low	Low	Low
Alcaraz et al	Low	Low	Low	Low	High	Unclear	Low
Balci et al	Low	Low	Unclear	Low	Low	Low	Unclear
Bolog et al	Low	Unclear	Low	Low	High	Unclear	Low
Brand et al	Low	Low	Low	High	High	Low	Low
Buscail et al	Low	Unclear	Low	Low	High	Unclear	Low
Catalano et al	Unclear	Low	Unclear	Low	Low	Low	Low
Chong et al	Low	Low	Low	Low	Low	Low	Low
Conwell et al	Low	Low	High	Low	Low	Low	Unclear
Dramaix et al	Low	Low	Low	Low	Low	Unclear	Low
Fusari et al	Unclear	Low	Low	Low	High	Low	Low
Gebel et al	Low	Low	Low	High	Low	Unclear	Low
Giovannini et al	Unclear	Unclear	Low	Low	High	Unclear	Unclear
Glasbrenner et al	Low	Low	Low	Low	High	Low	Low
Gmelin et al	Low	Low	Low	Low	Low	High	Unclear
Hellerhoff et al	Low	Low	Low	Low	Low	Low	Low
Imdahl et al	Low	Low	Unclear	Low	Low	Unclear	Low
Kremer et al	High	Unclear	Unclear	High	High	Unclear	Low
Lammer et al	Low	Low	Unclear	Low	Low	Unclear	Unclear
Lawson et al	Low	Low	Unclear	Low	Low	Low	Unclear
Lees et al	Low	Low	Low	High	Low	High	Low
Lin et al	High	Unclear	Low	Low	Low	Unclear	Low
Nattermann et al	Unclear	Low	Low	Low	High	Unclear	Low
Pamos et al	Low	Low	Low	Low	High	Unclear	Low
Parsi et al	Low	Low	Low	Low	Low	Low	Low
Pistolesi et al	Unclear	Low	Low	Low	Low	Low	Low
Pungpapong et al	Low	Low	Low	Low	Low	Low	Low
Pungpapong et al	Low	Unclear	Unclear	Low	Low	Low	Low
Rudowicz Pietr-uszewska et al	Low	Unclear	Low	Low	High	Unclear	Unclear
Sai et al	High	Low	Low	Low	Low	Low	Low
Savarino et al	Unclear	Low	Low	Low	Low	Low	Low
Scarabino et al	Low	Unclear	Unclear	Low	High	Unclear	Unclear
Schlaudraff et al	Low	Unclear	Low	Low	Low	Low	Low
Stevens et al	Low	Low	Unclear	Low	Low	Low	Unclear
Sverko et al	Unclear	Unclear	Low	Low	Low	Unclear	Low
Swobodnik et al	Low	Low	Low	Low	Low	Low	Low
Tox et al	Low	Unclear	Unclear	Low	Low	Low	Low
Trikudanathan et al	Unclear	Low	Unclear	Low	High	Low	Low
Triller et al	Unclear	Low	Unclear	Low	Unclear	Unclear	Low
Wiersema et al	Unclear	Low	Unclear	Low	High	Low	Unclear
Zhang et al	High	Unclear	High	Low	Low	Unclear	High
Zuccaro et al	Unclear	Low	Unclear	Low	Low	Low	Unclear

Table 8

**Table 8:** GRADE scoring system

EUS									
Outcome	№ of studies (№ of patients)	Study design	Factors that may decrease quality of evidence					Effect per 1000 patients tested	Quality of evidence
			Risk of bias	Indirectness	Inconsistency	Imprecision	Publication bias	Pre-test probability of 47.2%	
True positives	16 (1249)	Cohort & case-control	Serious ^a^	Serious ^b^	Very serious ^c^	Very serious ^d^	NA	387 (335 to 425)	⨁◯◯◯ VERY LOW
False negatives								85 (47 to 137)	
True negatives	16 (1249)	Cohort & case-control	Serious ^a^	Serious ^b^	Serious ^c^	Serious ^d^	NA	480 (438 to 502)	⨁◯◯◯ VERY LOW
False positives								48 (26 to 90)	
ERCP									
Outcome	№ of studies (№ of patients)	Study design	Factors that may decrease quality of evidence					Effect per 1000 patients tested	Quality of evidence
			Risk of bias	Indirectness	Inconsistency	Imprecision	Publication bias	Pre-test probability of 42.6%	
True positives	11 (742)	Cohort & case-control	Not serious ^e^	Serious ^f^	Serious ^g^	Serious ^h^	NA	349 (324 to 371)	⨁◯◯◯ Very low
False negatives								77 (55 to 102)	
True negatives	11 (742)	Cohort & case-control	Not serious ^e^	Serious ^f^	Serious ^g^	Serious ^h^	NA	540 (499 to 563)	⨁◯◯◯ Very low
False positives								34 (11 to 75)	
MRCP									
Outcome	№ of studies (№ of patients)	Study design	Factors that may decrease quality of evidence					Effect per 1000 patients tested	Quality of evidence
			Risk of bias	Indirectness	Inconsistency	Imprecision	Publication bias	Pre-test probability of 28.9%	
True positives	14 (933)	Cohort & case-control-type studies	Serious ^i^	Serious ^j^	Serious ^k^	Very serious ^l^	NA	225 (199 to 246)	⨁◯◯◯ Very low
False negatives								64 (43 to 90)	
True negatives	14 (933)	Cohort & case-control-type studies	Serious ^i^	Serious ^j^	Serious ^k^	Not serious ^l^	NA	683 (640 to 697)	⨁◯◯◯ Very low
False positives								28 (14 to 71)	
CT									
Outcome	№ of studies (№ of patients)	Study design	Factors that may decrease quality of evidence					Effect per 1000 patients tested	Quality of evidence
			Risk of bias	Indirectness	Inconsistency	Imprecision	Publication bias	Pre-test probability of 38.4%	
True positives	10 (700)	Cohort & case-control	Serious ^m^	Serious ^n^	Serious ^o^	Very serious ^p^	NA	288 (253 to 319)	⨁◯◯◯ Very low
False negatives								96 (65 to 131)	
True negatives	10 (700)	Cohort & case-control	Serious ^m^	Serious ^n^	Serious ^o^	Serious ^p^	NA	561 (499 to 591)	⨁◯◯◯ Very low
False positives								55 (25 to 117)	
US									
Outcome	№ of studies (№ of patients)	Study design	Factors that may decrease quality of evidence					Effect per 1000 patients tested	Quality of evidence
			Risk of bias	Indirectness	Inconsistency	Imprecision	Publication bias	pre-test probability of 25.7%	
True positives	10 (1005)	Cohort & case-control	Serious ^q^	Serious ^r^	Serious ^s^	Very serious ^t^	NA	172 (136 to 200)	⨁◯◯◯ Very low
False negatives								85 (57 to 121)	
True negatives	10 (1005)	Cohort & case-control	Serious ^q^	Serious ^r^	Very serious ^s^	Serious ^t^	NA	728 (661 to 743)	⨁◯◯◯ Very low
False positives								15 (0 to 82)	

*NA* not available

^a^Risk of bias: based on QUADAS-2 risk of bias; 7 studies not serious, 9 studies serious

^b^Indirectness: based on QUADAS-2 applicability; 7 studies not serious, 9 studies serious

^c^Inconsistency: based on heterogeneity and visual inspection CIs

^d^Imprecision: based on study numbers and CIs of summary estimate (CIs 0–10 = not serious, 11–15 = serious, more than 15 = very serious)

^e^Based on QUADAS-2 risk of bias: 8 studies not serious, 3 studies serious

^f^Based on QUADAS-2 applicability: 6 studies not serious, 5 studies serious

^g^Based on heterogeneity and visual inspection CIs

^h^Based on study numbers and CIs of summary estimate (CIs 0–10 = not serious, 11–15 = serious, more than 15 = very serious)

^i^Risk of bias: based on QUADAS-2 risk of bias; 7 studies not serious, 5 studies serious, 1 study very serious

^j^Indirectness: based on QUADAS-2 applicability; 6 studies not serious, 8 studies serious

^k^Inconsistency: based on heterogeneity and visual inspection CIs

^l^Imprecision: based on study numbers and CIs of summary estimate (CIs 0–10 = not serious, 11–15 = serious, more than 15 = very serious)

^m^Risk of bias: based on QUADAS-2 risk of bias; 5 studies not serious, 5 studies serious

^n^Indirectness: based on QUADAS-2 applicability; 6 studies not serious, 4 studies serious

^o^Inconsistency: based on heterogeneity and visual inspection CIs

^p^Imprecision: based on study numbers and CIs of summary estimate (CIs 0–10 = not serious, 11–15 = serious, more than 15 = very serious)

^q^Risk of bias: based on QUADAS-2 risk of bias; 6 studies not serious, 3 studies serious, 1 study very serious

^r^Indirectness: based on QUADAS-2 applicability; 5 studies not serious, 5 studies serious

^s^Inconsistency: based on heterogeneity and visual inspection CIs

^t^Imprecision: based on study numbers and CIs of summary estimate (CIs 0–10 = not serious, 11–15 = serious, more than 15 = very serious)

Table 9

**Table 9:** GRADE characteristics for each study

Modality	Name first author	Risk of bias	Indirectness	Inconsistency	Imprecision	Publication bias
EUS	Albashir et al	Low	Low	Sensitivity: very serious Specificity: serious	Sensitivity: very serious Specificity: serious	Not assessed
	Brand et al	Serious	Serious			
	Buscail et al	Serious	Serious			
	Catalano et al	Low	Low			
	Chong et al	Low	Low			
	Conwell et al	Serious	Serious			
	Giovannini et al	Serious	Serious			
	Glasbrenner et al	Low	Low			
	Lin et al	Serious	Serious			
	Nattermann et al	Low	Serious			
	Pungpapong et al	Low	Low			
	Pungpapong et al	Low	Low			
	Stevens et al	Serious	Serious			
	Tox et al	Serious	Low			
	Trikudanathan et al	Serious	Serious			
	Wiersema et al	Serious	Serious			
ERCP	Adamek et al	Low	Low	Sensitivity: serious Specificity: serious	Sensitivity: serious Specificity: serious	Not assessed
	Buscail et al	Low	Serious			
	Gebel et al	Low	Low			
	Glasbrenner et al	Low	Low			
	Gmelin et al	Low	Serious			
	Lammer et al	Serious	Serious			
	Lawson et al	Low	Low			
	Parsi et al	Low	Low			
	Scarabino et al	Serious	Serious			
	Swobodnik et al	Low	Low			
	Triller et al	Serious	Serious			
MRCP	Adamek et al	Low	Low	Sensitivity: serious Specificity: serious	Sensitivity: very serious Specificity: not serious	Not assessed
	Alcaraz et al	Low	Serious			
	Balci et al	Serious	Serious			
	Bolog et al	Serious	Serious			
	Fusari et al	Low	Low			
	Hellerhoff et al	Low	Low			
	Pamos et al	Low	Serious			
	Pungpapong et al	Low	Low			
	Rudowicz-Pietruszewska	Serious	Serious			
	Sai et al	Serious	Low			
	Schlaudraff et al	Low	Low			
	Sverko et al	Serious	Serious			
	Zhang et al	Very serious	Serious			
	Zuccaro et al	Serious	Serious			
CT	Buscail et al	Low	Serious	Sensitivity: serious Specificity: serious	Sensitivity: very serious Specificity: serious	Not assessed
	Dramaix et al	Low	Low			
	Fusari et al	Low	Low			
	Gmelin et al	Low	Serious			
	Imdahl et al	Serious	Low			
	Lammer et al	Serious	Serious			
	Pistolesi et al	Low	Low			
	Savarino et al	Serious	Low			
	Scarabino et al	Serious	Serious			
	Swobodnik et al	Low	Low			
US	Buscail et al	Low	Serious	Sensitivity: serious Specificity: very serious	Sensitivity: very serious Specificity: serious	Not assessed
	Dramaix et al	Low	Low			
	Gebel et al	Low	Low			
	Gmelin et al	Low	Serious			
	Kremer et al	Very serious	Serious			
	Lawson et al	Low	Low			
	Lees et al	Serious	Low			
	Lin et al	Serious	Serious			
	Scarabino et al	Serious	Serious			
	Swobodnik et al	Low	Low			

Table 10

**Table 10:** Diagnostic characteristics for each study

Study	Sensitivity	Specificity	Accuracy	PPV	NPV	TP	TN	FP	FN
Adamek et al	MRCP: 88%, ERCP: 90%	MRCP: 94%. ERCP: 91%	MRCP: 91% ERCP: 90%	MRCP: 93%, ERCP:90%	MRCP: 90%, ERCP: 91%	MRCP:50 ERCP: 51	MRCP: 63 ERCP: 61	MRCP: 4 ERCP: 6	MRCP: 7 ERCP: 6
Albashir et al	84%	100%	87%	100%	57%	16	4	0	3
Alcaraz et al	50%	99%	94%	80%	95%	4	72	1	4
Balci et al	82%	63%	70%	56%	86%	9	12	7	2
Bolog et al	90%	98%	95%	90%	98%	14	86	2	1
Brand et al	42%	96%	84%	71%	86%	10	87	4	14
Buscail et al	US: 58%,CT: 75%, ERCP: 74%, EUS: 88%	US: 75%, CT: 95%, ERCP: 100%, EUS: 100%	US: 65%, CT: 81%, ERCP: 82%, EUS: 92%	US: 87%, CT: 97%, ERCP: 100%, EUS: 100%	US: 44%, CT: 61%, ERCP: 62%, EUS: 78%	US: 26, CT: 33, ERC: 33, EUS: 39	US: 14, CT: 17, ERCP: 18, EUS: 18	US: 4, CT: 1, ERCP: 0, EUS: 0	US: 18, CT: 11, ERCP: 11, EUS: 5
Catalano et al	84%	98%	91%	97%	87%	32	41	1	6
Chong et al	83%	80%	83%	98%	69%	53	5	1	11
Conwell et al	26%	100%	50%	100%	39%	10	18	0	28
Dramaix et al	CT: 60%, US: 60%	CT: 100% US: 95%	CT: 86% US: 82%	CT: 100%, US: 90%	CT: 76%, US: 76%	CT: 11, US: 11	CT: 32, US: 30	CT: 0, US: 2	CT: 7, US: 7
Fusari et al	CT: 88%, MRI: 88%	CT: 100%, MRI:100%,	CT: 98%, MRI: 98%	CT: 100%, MRI: 100%	CT: 97%, MRI: 97%	MRI: 7, CT: 7	MRI: 32, CT: 32	MRI: 0, CT: 0	MRI: 1, CT: 1
Gebel et al	US: 82%, ERP: 56%	US: 97%, ERP: 97%	US: 91%, ERP: 82%	US:95%, ERP: 90%	US: 89%, ERP: 80%	US: 18, ERP: 9	US: 33, ERP: 28	US: 1, ERP: 1	US: 4, ERP: 7
Giovannini et al	94%	56%	81%	80%	83%	16	5	4	1
Glasbrenner et al	EUS: 93%, ERCP: 88%	EUS: 78%, ERCP: 82%	EUS: 85%, ERCP: 85%	EUS: 79%, ERCP: 82%	EUS: 92%, ERCP: 88%	EUS: 38, ERCP: 36	EUS: 34, ERCP: 36	EUS: 10, ERCP: 8	EUS: 3, ERCP: 5
Gmelin et al	US: 68%, CT: 84%, ERCP: 89%	US: 100%, CT: 91%, ERCP: 91%	US: 85%, CT: 89%, ERCP: 90%	US: 100%, CT: 89%, ERCP: 89%	US: 79%, CT: 87%, ERCP: 91%	US: 13, CT: 16, ERCP: 17	US: 22, CT: 20, ERCP: 20	US: 0, CT: 2, ERCP: 2	US: 6, CT: 3. ERCP: 2
Hellerhoff et al	MRI: 77%, sMRI: 89%	MRI: 100%, sMRI:100%	MRI 94%, sMRI: 97%	MRI: 100%, sMRI: 100%	MRI: 92%, sMRI: 96%	MRI: 20, sMRI: 23	MRI: 69, sMRI: 69	MRI: 0, sMRI: 0	MRI: 6, sMRI: 3
Imdahl et al	58%	91%	83%	70%	85%	7	33	3	5
Kremer et al	67%	99%	94%	89%	95%	42	378	5	21
Lammer et al	ERCP: 85%, CT: 64%	ERCP: 97%, CT: 85%	ERCP: 93%, CT: 78%	ERCP: 94%, CT: 71%	ERCP: 92%, CT: 81%	ERCP: 33, CT: 25	ERCP: 66, CT: 58	ERCP: 2, CT: 10	ERCP: 6, CT: 14
Lawson et al	US: 38%, ERCP: 73%	US: 100%, ERCP: 98%	US: 79%, ERCP: 98%	US: 100%, ERCP: 95%	US: 75%, ERCP: 87%	US: 10, ERCP: 19	US: 49, ERCP: 48	US: 0, ERCP: 1	US: 16, ERCP: 7
Lees et al	100%	97%	98%	91%	100%	20	76	2	0
Lin et al	US: 86%, EUS: 100%	US: 100%, EUS: 100%	US: 97%, EUS: 100%	US: 100%, EUS: 100%	US: 96%, EUS: 100%	US: 6, EUS: 7	US: 26, EUS: 26	US: 0, EUS: 0	US: 1, EUS: 0
Nattermann et al	98%	57%	75%	65%	97%	50	36	27	1
Pamos et al	80%	100%	98%	100%	97%	4	36	0	1
Parsi et al	71%	91%	77%	94%	59%	17	10	1	7
Pistolesi et al	58%	81%	74%	58%	81%	18	56	13	13
Pungpapong et al	71%	88%	80%	84%	77%	27	36	5	11
Pungpapong et al	EUS: 93%, MRCP: 65%	EUS: 93%, MRCP: 90%	EUS: 93%, MRCP: 80%	EUS: 90%, MRCP: 81%	EUS: 95%, MRCP: 79%	EUS: 37, MRCP: 26	EUS: 55, MRCP: 53	EUS: 4, MRCP: 6	EUS: 3, MRCP: 14
Rudowicz-Pietruszewska et al	100%	100%	100%	100%	100%	9	79	0	0
Sai et al	60%	79%	68%	77%	60%	10	9	3	6
Savarino et al	90%	59%	76%	73%	83%	53	29	20	6
Scarabino et al	ERCP: 83%, US: 42%, CT: 100%	ERCP: 67%, US: 34%, CT: 70%	ERCP: 70%, US: 35%, CT: 76%	ERCP: 37%, US: 13%, CT: 44%	ERCP: 94%, US: 71%, CT: 100%	ERCP: 10, US: 5, CT: 12	ERCP: 34, US: 17, CT: 36	ERCP: 17, US: 34, CT: 15	ERCP: 2, US: 7, CT: 0
Schlaudraff et al	MRCP: 67%, sMRCP: 73%	MRCP: 93%, sMRCP: 96%	MRCP: 89%, sMRCP: 93%	MRCP: 63%, sMRCP: 78%	MRCP: 95%, sMRCP: 95%	MRCP: 6, sMRCP: 7	MRCP: 49, sMRCP: 51	MRCP: 4, sMRCP: 2	MRCP: 3, sMRCP: 2
Stevens et al	Radial: 68%, Linear: 44%	Radial: 95% Linear: 95%	Radial: 84% Linear: 74%	Radial: 90%, Linear: 86%	Radial: 81%, Linear: 71%	28	56	3	13
Sverko et al	79%	93%	86%	92%	82%	11	14	1	3
Swobodnik et al	US: 52%, CT: 74%, ERCP: 93%	US: 100%, CT: 98%, ERCP: 100%	US: 84%, CT: 90%, ERCP: 98%	US: 100%, CT: 95%, ERCP: 100%	US: 81%, CT: 88%, ERCP: 96%	US: 14, CT: 20, ERCP: 25	US: 54, CT: 53, ERCP: 54	US: 0, CT: 1, ERCP: 0	US: 13, CT: 7, ERCP: 2
Tox et al	77%	75%	76%	66%	84%	50	80	26	15
Trikudanathan et al	61%	75%	63%	92%	29%	34	9	3	22
Triller et al	82%	85%	83%	82%	85%	9	11	2	2
Wiersema et al	80%	86%	84%	83%	84%	24	32	5	6
Zhang et al	92%	75%	84%	81%	88%	22	15	5	2
Zuccaro et al	MRCP: 46%, sMRCP: 46%	MRCP:85%, sMRCP: 68%	MRCP: 70%, sMRCP: 59%	MRCP: 68%, sMRCP: 50%	MRCP: 70%, sMRCP: 65%	MRCP: 13, sMRCP: 13	MRCP: 35, sMRCP: 28	MRCP: 6, sMRCP: 13	MRCP: 15, sMRCP: 15

*PPV* positive predictive value, *NPV* negative predictive value, *TP* true positive, *TN* true negative, *FP* false positive, *FN* false negative

Table 11

**Table 11:** Imaging characteristics for each study

Magnetic resonance imaging (MRI)							
Study	Year	Magnetic field	Coil type	Contrast	Secretin enhancement	Sequence	Scoring criteria
Adamek et al	2000	1.0 T	Body coil	No	No	T2	Size of common bile and pancreatic duct, the nature and degree of pancreatic duct obstruction, and accuracy in diagnosing pathological findings
Alcaraz et al	2000	1.5 T	NA	No	No	T2 (HASTE & RARE)	NA
Balci et al	2006	1.5 T	Four-element quadrature phased-array surface coil	IV	No	T1, T2	Increased arterial enhancement pattern, normal gland size and normal ductal morphology (Cambridge classification)
Bolog et al	2004	1.0 T	Synergy body coil	NA	No	T1, T2	NA
Fusari et al	2010	1.5 T	Phased-array synergy body coil	Oral	No	T1, T2	1–5 score to identify pancreatic masses (definite benign = 1, probably benign = 2 etc.)
Hellerhoff et al	2002	1.5 T	Phased-array synergy surface coil	Oral	No	T2	Cambridge classification
Pamos et al	1998	1.5 T	Body coil	NA	No	T2	NA
Pungpapong et al	2007	1.5 T	Phased-array surface coil	IV/Oral	No	T1, T2, T2 (HASTE)	Presence of 1 or more of the following features: main pancreatic duct dilatation in absence of structural obstruction, dilated side branches, intraductal stones, ductal irregularity, reduced T1 signal intensity, atrophy of pancreatic parenchyma and reduced secretory response to secretin administration
Rudowicz-Pietruszewska et al	2002	0.5 T	Body coil	NA	No	T2	NA
Schlaudraff et al	2008	1.0 T	Dedicated quadrature torso phased-array coil	NA	No	T2, T2 (HASTE)	Pancreatic duct stenosis/dilatation, side branch stenosis/dilatation, pseudocysts, extrapancreatic abscess. Based on observers’ judgement
Sverko et al	2011	1.0 T	NA	IV	No	T1, T2 (HASTE)	NA
Zhang et al	2003	1.5 T	NA	IV	No	T1	Signal intensity by gadolinium (presence of SIR less than 1.73 in the arterial phase)
Zuccaro et al	2009	NA	Phased array-torso coil	IV	No	T1, T2, T2 (HASTE)	Mild CP: secretin-induced T2 intensity significantly reduced; side branch ectasia, mild ductal dilatation. Moderate CP: abnormal enhancement pattern on T1 after gadolinium administration. Severe CP: atrophy or diffuse/focal enlargement of the gland, calcification, chronic pseudocysts
Secretin-enhanced magnetic resonance imaging (sMRI)							
Hellerhoff et al	2002	1.5 T	Synerge phased-array surface coil	IV	Yes	T2	Cambridge classification
Sai et al	2008	1.5 T	Phased-array multi coil	IV	Yes	NA	Cambridge classification
Schlaudraff et al	2008	1.0 T	Dedicated quadrature phased-array torso coil	NA	Yes	T2, T2 (HASTE)	Pancreatic duct stenosis/dilatation, side branch stenosis/dilatation, pseudocysts, extrapancreatic abscess. Based on observers’ judgement
Zuccaro et al	2009	NA	Phased-array torso coil	IV	Yes	T1, T2, T2 (HASTE)	Mild CP: secretin-induced T2 intensity significantly reduced; side branch ectasia, mild ductal dilatation. Moderate CP: abnormal enhancement pattern on T1 after gadolinium administration. Severe CP: atrophy or diffuse/focal enlargement of the gland, calcification, chronic pseudocysts
Ultrasonography (US)							
Study	Year	Transducer	Scoring criteria				
Buscail et al	1995	NA	NA				
Dramaix et al	1980	Unirad/Kretz combison 200	NA				
Gebel et al	1985	ADR 2130 Imager 2380 Sonoline 8000	Duct abnormalities				
Gmelin et al	1981	Sono fluoroskop 1, unirad model 849	Criteria for PC, CP and normal pancreas were extracted from literature				
Kremer et al	1977	NA	Rettenmaier specified examination technique				
Lawson et al	1978	13-mm diameter 3.5 Mhz/ 13 or 19-mm diameter 2.25 Mhz	Identification of a mass, pseudocyst or generalized glandular enlargement with abnormal parenchymal echogenicity				
Lees et al	1979	2.5 Mhz	Appearance of pancreatic parenchyma and duct system/size and shape of the pancreas and from previous reports				
Lin et al	1989	SAL-90A 3.75 Mhz	NA				
Scarabino et al	1989	NA	NA				
Swobodnik et al	1983	Siemens imager 2300 linear array	Organ enlarged or atrophic dense structure, areas of scars or calcification (more echogenic), sonolucent areas only during acute inflammation, dilatation of the pancreatic duct system, symmetric contours, no smooth outlines				
Computed tomography (CT)							
Study	Year	Scanner	Contrast	Scoring criteria			
Buscail et al	1995	NA	NA	NA			
Dramaix et al	1980	OHIO nuclear - Delta Slan 50FS	Oral/IV	NA			
Fusari et al	2010	Marconi MX8000 (four-detector row)	IV	1–5 score to identify pancreatic masses (definite benign = 1, probably benign = 2 etc.)			
Gmelin et al	1981	NA	NA	Criteria for PC, CP and normal pancreas were extracted from literature			
Imdahl et al	1999	Somatom Plus 4 helical scanner	IV	NA			
Lammer et al	1980	EMI-5005	Oral	3 stadia typical for CP			
Pistolesi et al	1981	Ohio-Nuclear Delta 50 scanner	NA	Overall enlargement of the pancreas or calcifications			
Savarino et al	1980	EMI-5005	Oral	Parenchymal atrophy, pancreatic calcifications, pseudocysts or abscesses			
Scarabino et al	1989	NA	NA	NA			
Swobodnik et al	1983	General Electric CT-T8800	Oral/IV	Atrophy of the organ (during acute inflammation: segmental enlargement) during acute phase; segments without clear outlines, cysts or calcifications and dense structure			
Endoscopic ultrasonography (EUS)							
Study	Year	Scanner	Transducer	MHz	Scoring criteria		
Albashir et al	2010	NA	NA	NA	9 features; >4 diagnostic for CP		
Brand et al	2000	Olympus GF-UM 3/GF-UM 20/GF-UM 200	Radial	NA	Own criteria (increased parenchymal lobulations, calcification and/or ductal changes or focal lesion)		
Buscail et al	1995	Olympus EU-M3	NA	7.5/12 MHz	NA		
Catalano et al	1998	Olympus EU-M3/EU-M20	NA	7.5/12 MHz	Wiersema criteria (11 features), own classification system		
Chong et al	2007	Olympus EU-M20/GF-UM130/GF-UM160/GF-UC30P/GF-UC140P/GF-UCT140	Radial	NA	9 features; >3 diagnostic for CP		
Conwell et al	2007	NA	NA	NA	9 features; >3 diagnostic for CP		
Giovannini et al	1994	Pentax FG-32-UA	Linear	NA	NA		
Glasbrenner et al	2000	Olympus EU-M20	Radial	7.5/12 MHz	Wiersema criteria (11 features)		
Lin et al	1989	Olympus GF-EUM 2/GF-UM2	Radial	7.5 MHz	NA		
Nattermann et al	1993	Olympus GF-UM-3/EU-M3	NA	7.5/12 MHz	NA		
Pungpapong et al	2007	Olympus GF-UE160-AL5/GF-UC140P	Radial & linear	NA	MST criteria; >4 features diagnostic for CP		
Pungpapong et al	2007	Olympus GF-UC140P/ UCT140-AL5	Linear	7.5 MHz	MST criteria; >4 features diagnostic for CP		
Stevens et al	2009	Olympus GF-UM-130/GF-UE-160/GF-UC-160P-OL5	Radial & linear	NA	9 features; >4 diagnostic for CP		
Tox et al	2007	Olympus GF-UM20, Pentax EG-3620-UR/EG-3830-UT	NA	NA	Own criteria		
Trikudanathan	2016	Olympus	Linear	7.5 MHz	Wiersema criteria (11 features) >4 is CP		
Wiersema et al	1993	Olympus EU-M3/EU-M20	NA	NA	Wiersema criteria (11 features) >3 is CP		
Endoscopic retrograde cholangiopancreatography (ERCP)							
Study	Year	Technical features	Scoring criteria				
Adamek et al	2000	NA	NA				
Buscail et al	1995	NA	Own criteria (normal/moderate changes (3 abnormal side branches and normal main duct)/marked changes (side and main duct abnormalities))				
Gebel et al	1995	NA	Deyhle criteria				
Glasbrenner et al	2000	Olympus	Cambridge classification				
Gmelin et al	1981	NA	Criteria according to references				
Lammer et al	1980	Olympus JFB	Loffler criteria				
Lawson et al	1978	NA	Criteria according to references				
Parsi et al	2008	NA	Cambridge classification				
Scarabino et al	1989	NA	NA				
Swobodnik et al	1983	Olympus JFB-2/3	Own criteria (variation in diameter of the main duct in the whole organ (exception: segmental pancreatitis), cystic dilatation of side branches, kinking of the duct stones in canalicular structures, distension of the main duct)				
Triller et al	1975	NA	NA				

*NA* not available
